# Chemical Exposures Affect Innate Immune Response to SARS-CoV-2

**DOI:** 10.3390/ijms222212474

**Published:** 2021-11-19

**Authors:** Olatunbosun Arowolo, Leonid Pobezinsky, Alexander Suvorov

**Affiliations:** 1 Department of Environmental Health Sciences, School of Public Health and Health Sciences, University of Massachusetts, 686 North Pleasant Street, Amherst, MA 01003, USA; oarowolo@umass.edu; 2 Department of Veterinary and Animal Sciences, College of Natural Sciences, University of Massachusetts, 661 North Pleasant Street, Amherst, MA 01003, USA; lpobezinsky@umass.edu

**Keywords:** COVID-19, SARS-CoV-2, in silico, toxicity, xenobiotics, IFN signaling

## Abstract

Severe outcomes of COVID-19 are associated with pathological response of the immune system to the SARS-CoV-2 infection. Emerging evidence suggests that an interaction may exist between COVID-19 pathogenesis and a broad range of xenobiotics, resulting in significant increases in death rates in highly exposed populations. Therefore, a better understanding of the molecular basis of the interaction between SARS-CoV-2 infection and chemical exposures may open opportunities for better preventive and therapeutic interventions. We attempted to gain mechanistic knowledge on the interaction between SARS-CoV-2 infection and chemical exposures using an in silico approach, where we identified genes and molecular pathways affected by both chemical exposures and SARS-CoV-2 in human immune cells (T-cells, B-cells, NK-cells, dendritic, and monocyte cells). Our findings demonstrate for the first time that overlapping molecular mechanisms affected by a broad range of chemical exposures and COVID-19 are linked to IFN type I/II signaling pathways and the process of antigen presentation. Based on our data, we also predict that exposures to various chemical compounds will predominantly impact the population of monocytes during the response against COVID-19.

## 1. Introduction

In December 2019, a novel infectious disease was recognized [1]. The disease was named coronavirus disease 2019 (COVID-19) and it is known now to be caused by coronavirus SARS-CoV-2. As of 20 March 2021, the COVID-19 pandemic has recorded almost 122 million cases with 2,694,094 mortalities spanning 223 countries of the world [2]. Recently, many SARS-CoV-2 vaccines have been developed, but global morbidity and mortality due to COVID-19 remain substantial. Additionally, the evolution of SARS-CoV-2 challenges existing vaccines and threatens the global population with new virus variants. Therefore, a better understanding of factors affecting COVID-19-caused morbidity and mortality is needed.

An emerging body of studies demonstrates that diverse chemical exposures can increase COVID-19 severity. For example, a 1 µg/m^3^ increase in particulate matter (PM2.5) in air is responsible for a greater than 8% increase in the death rate from COVID-19 [3]. Exposure to air pollutants is the major culprit behind the significant COVID-19 mortalities in different countries of the world [4]. The major types of air pollutants that can increase the COVID-19 severity include ozone, carbon monoxides, sulphur dioxides, lead, volatile organic compounds, particulate matter (PM_2.5_ and PM_10_), and nitrogen oxides [5,6,7,8].

Besides air pollutants, several other chemicals have also been associated with COVID-19 severity. For example, one study identified significant associations between COVID-19 infection fatality rates and toxic industrial chemicals in the United States [9]. Increased severity of COVID-19 was also reported owing to the use of sanitizers and disinfecting chemicals [9,10,11,12]. Plasma levels of perfluorinated compounds were also associated with an increased risk of COVID-19 [13]. It has also been hypothesized that neuroinflammation induced by environmental chemicals may increase neurological symptoms in COVID-19 patients [14].

Thus, existing data demonstrate that a variety of chemical exposures increase the severity of COVID-19. A better understanding of the molecular basis of interaction between SARS-CoV-2 infection and chemical exposures may open opportunities for better preventive and therapeutic interventions. Analysis of the interaction of SARS-CoV-2 infection with all chemicals to which humans are exposed is, however, challenging, if not an impossible task.

In our recent study, we attempted to identify genes and molecular pathways most sensitive to chemical exposures using an unbiased big-data approach by overlying transcriptomic datasets from 2169 individual studies [15]. This unbiased analysis demonstrated that immune response pathways, including interferon signaling and cytokine signaling, are pathways highly sensitive to chemical exposures [15]. Immunotoxic effects of many environmental and occupational chemical compounds have long been recognized [16]. Emerging evidence from experimental and population studies demonstrates the ability of a broad range of xenobiotics to interact with immunological pathways [17,18,19,20]. Thus, we hypothesize that specific immune response mechanisms may be synergistically affected by chemical exposures and SARS-CoV-2, resulting in increased severity of the disease.

Emerging data on SARS-CoV-2 indicate that the disease severity is linked to a highly dysregulated innate immune response characterized by a delayed interferon response and exuberant inflammatory response [21,22]. Studies demonstrated that SARS-CoV-2 blunts the production of type I interferons (IFN), molecules representing the first line of innate antiviral defense [21,23]. Additionally, SARS-CoV-2 infection can lead to excessive production of pro-inflammatory cytokines (cytokine storm) [24,25]. These mechanisms may be linked causally, as a suppressed IFN response results in excessive production of proinflammatory cytokines, resulting in severe acute respiratory syndrome [21,26].

To test the hypothesis that SARS-CoV-2 and a broad range of xenobiotics may impair similar immunological mechanisms, we used the in silico approach to identify genes and molecular pathways affected by both chemical exposures and SARS-CoV-2 in major types of human immune cells. Our research predicts that chemical exposures and SARS-CoV-2 infection synergistically affect IFN type I/II signaling pathways and the process of antigen presentation.

## 2. Results

**Figure 1:**
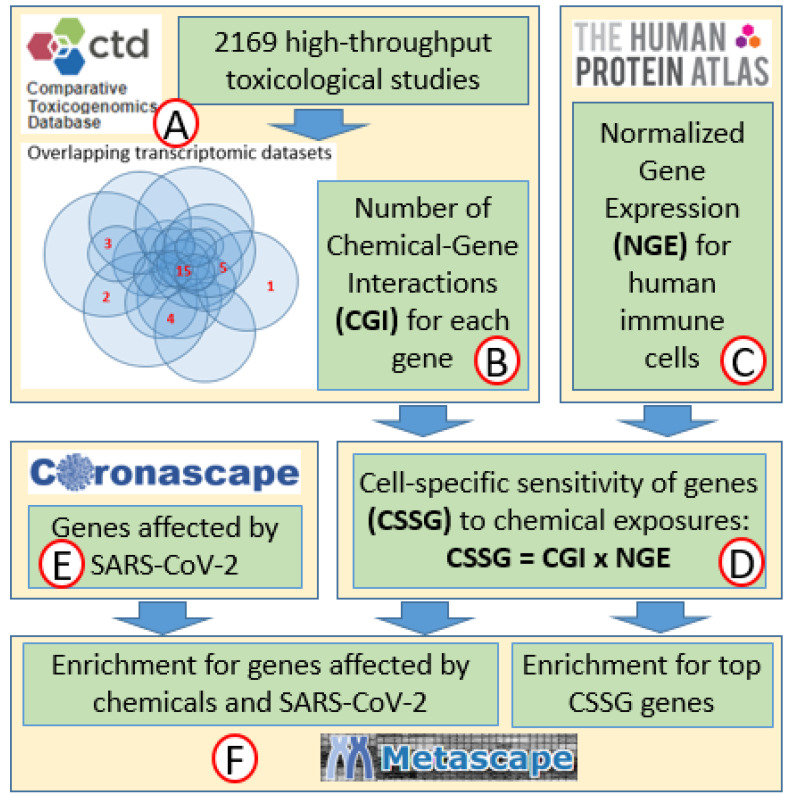
Flow chart of the study approach: transcriptomic information from 2169 studies was extracted from the Comparative Toxicogenomic Database (**A**) and used to calculate the sensitivity of every gene to chemical exposures (**B**) [15]. Information on the expression of every gene in different types of immune cells was extracted from the Human Protein Atlas (**C**) and used to normalize the sensitivities of genes to chemical exposures for the level of expression in different cell types (**D**). Information on changes in gene expression in different immune cells in response to SARS-CoV-2 was extracted from Coronoscape (**E**). Metascape (**F**) was used to identify molecular pathways enriched with genes highly sensitive to chemical exposures in immune cells and genes affected in these cells by both chemical exposures and SARS-CoV-2.

According to our hypothesis, specific immune response mechanisms may be synergistically affected by chemical exposures and SARS-CoV-2, resulting in increased severity of the disease. To test this hypothesis, we first used data on the sensitivity of mammalian genes to chemical exposures identified in our previous research [15,27] by overlaying transcriptomic datasets from many individual toxicological genomic studies. This allowed the identification of genes that respond most often to a variety of chemical exposures (Figure 1). At that step, the sensitivity of a gene to chemical exposure was expressed as the number of chemical–gene interactions (CGIs). CGI reflects the number of individual toxicological studies in which the gene of interest was affected by exposure.

Genes sensitive to chemical exposures are not equally expressed in different cell types. If some cells have very low levels of expression of chemically sensitive genes in normal physiological conditions, then it is reasonable to assume that chemical exposures will not significantly alter the physiology of these cells. On the contrary, cells expressing highly chemically sensitive genes will respond to chemical exposure by the significant change in their physiology. Thus, to model cell response to xenobiotics, we need to understand the levels of expression of chemically sensitive genes in this specific cell type. Following this logic, we generated lists of genes with their chemical sensitivity values adjusted for the level of expression in different human immune cells. We call these adjusted values cell-specific sensitivity of genes (CSSG).

At the next step, for different immune cells, we compared the lists of genes with the highest CSSG values with genes affected by SARS-CoV-2 in the same cell types, in order to identify synergistic effects of both stressors. Finally, we used genes with the highest CSSG values as well as genes affected by both chemical exposures and SARS-CoV-2 in Metascape enrichment analysis to identify molecular mechanisms most sensitive to xenobiotics and both stressors in each cell type.

Below, we provide an analysis of molecular pathways enriched with the top CSSG genes and with genes that are sensitive to both chemical exposures and SARS-CoV-2.

### 2.1. Monocytes

#### 2.1.1. Top CSSG for Monocytes

The list of top genes with the highest CSSG values in monocytes includes 409 genes (Supplemental File 1). Top Metascape enriched biological categories affected by chemical exposures are involved in the regulation of immune response, including cytokine signaling, degranulation and migration, phagocytosis, VEGFA-VEGFR2 signaling pathway, hemostasis, apoptosis, and response to toxic substance (Table 1, Supplemental File 2).

#### 2.1.2. Genes Affected by Chemicals and COVID-19

**Figure 2:**
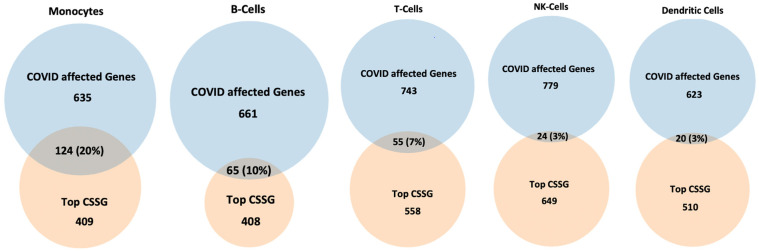
Overlap in genes affected by chemical exposures and SARS-CoV-2 infection in different types of human immune cells.

We identified 635 genes affected by COVID-19. Out of these 635 genes, 124 genes (20%) overlapped with top monocyte-specific sensitive genes (Figure 2). The top overlapping genes include, for example, genes encoding cell cycle progression and differentiation (S100A8, S100A9); genes involved in the suppression of apoptosis (PLAC8); several EEF genes serving a broad range of functions in monocytes such as protein synthesis and delivery of aminoacyl tRNA to the ribosome; CD14 and IFITM3 involved in response to bacterial and viral infections, respectively; PABPC1 involved in ribosome recruitment and translation initiation; and NAPILI, playing an important role in DNA replication (Table 2). Metascape enrichment analysis showed that overlapping genes are involved in essential functions of the immune system (cytokine signaling, antigen processing and presentation, positive regulation of reactive oxygen species, metabolic processes, platelet degranulation, and response to bacterial and viral infections), respiratory diseases (pertussis, neutrophil degranulation), VEGFA-VEGFR2 signaling pathway, cell regulation, and death (apoptotic signaling pathway, I-kappaB kinase/NF-kappaB signaling) (Table 3, Supplemental File 3). 

### 2.2. NK Cell

#### 2.2.1. Top CSSG for NK-Cells

Top CSSG genes in NK cells include 649 genes (Supplemental File 1). Top Metascape enriched biological categories affected by chemical exposures are involved in the regulation of protein synthesis and processing (mRNA catabolism and processing, protein processing in endoplasmic reticulum, protein folding, protein catabolism, and proteolysis), immune response (cytokine signaling, VEGFA-VEGFR2 signaling, leukocyte activation, interferon signaling, and hemostasis), and mitochondria functions (mitochondrion organization, aerobic respiration, and the oxidation-reduction process) (Table 1, Supplemental File 2).

#### 2.2.2. Genes Affected by Chemicals and COVID-19 

A total of 779 genes were affected by COVID-19 in NK cells. Out of these, 24 genes (3%) overlapped with the list of top NK-cell specific chemically sensitive genes (CSSG) (Figure 2). The top overlapping genes include, for example, genes encoding interferons—antiviral signaling molecules (ISGI5 and ISG20), proteins involved in cytokine signal transduction (STAT1 and JAK1), antimicrobial protein of cytotoxic granules of NK-cells (GNLY), proteins involved in the regulation of viral gene expression (SP100), beta-actin involved in cell motility (ACTB), and proteins involved in the regulation of protein synthesis (TPT1, EEF2, and PABPC1) (Table 2). Metascape enrichment analysis revealed that overlapping genes are involved in the VEGFA-VEGFR2 signaling pathway, immune responses (interferon signaling, antigen processing and presentation, and cytotoxicity), and apoptosis (Table 3, Supplemental File 3).

### 2.3. T-Cells

#### 2.3.1. Top CSSG for T-Cells

A total of 558 genes were identified as top CSSG genes for T-cells (Supplemental File 1). Top Metascape enriched biological categories affected by chemical exposures are involved in the regulation of protein synthesis and processing (ribosome, protein folding, protein processing in endoplasmic reticulum, and others), immune response (cytokine signaling, VEGFA-VEGFR2 signaling, leukocyte activation, and response to viral infection), response to stress (response to toxic substance, transcriptional regulation by TP53, and regulation of cellular stress response), and apoptosis (Table 1, Supplemental File 2).

#### 2.3.2. Genes Affected by Chemicals and COVID-19

A total of 743 genes were affected by COVID-19. Out of these, 55 genes (7.4%) overlapped with top T-cell-specific sensitive genes (CSSG) (Figure 2). The top overlapping genes include, for example, genes involved in antiviral interferon signaling cascade (MX1 and ISGI5); genes involved in lymphocytes’ development, proliferation, and survival (IL7R and PIM1); genes involved in protein synthesis (EEF1A1, EEF2, and PABPC1); and STAT1, involved in the response to cytokines and growth factors (Table 2). Metascape analysis showed that the top overlapping genes are involved in immune response (cytokine signaling and production, response to interferon-gamma, antigen processing and presentation, I-kappaB kinase/NF-kappaB signaling, and P2X7 receptor signaling complex), hemopoiesis, chaperone-mediated autophagy, and cell cycle/apoptosis regulation (MYC signaling) (Table 3, Supplemental File 3).

### 2.4. B-Cells

#### 2.4.1. Top CSSG for B-Cells

The shortlist of the top CSSG genes for B-cells included 408 genes (Supplemental File 1). Top Metascape biological categories affected by chemical exposures are involved in protein synthesis (ribosome, ribosome assembly, regulation of translation, and others) and processing (protein ubiquitination, protein folding, and response to topological incorrect proteins), DNA biosynthesis, immune response (leucocyte activation, cytokine signaling, and VEGFA-VEGFR2 signaling), hemopoiesis, apoptotic signaling, and response to toxic substances (Table 1, Supplemental File 2).

#### 2.4.2. Genes Affected by Chemicals and COVID-19

A total of 660 genes were affected by COVID-19. Out of these, 65 genes (9.8%) overlapped with the top B-cell-specific chemically sensitive genes (CSSG) (Figure 2). The top overlapping genes include, for example, genes encoding for chaperones (HSPA5, PD1A4, PD1A6, CALR, and CD74); genes involved in the development and differentiation of B-cells into plasma cells (MS4A1); genes encoding components of B lymphocyte antigen receptor (CD79A and CD79B); and JCHAIN, a protein component of the antibodies IgM and IgA (Table 2). Metascape analysis showed that the top overlapping genes are involved in translation, protein folding, different components of immune system development, and immune response, including myeloid cell differentiation B-cell activation; cytokine signaling; antigen processing and presentation; I-kappaB kinase/NF-kappaB signaling; VEGFA-VEGFR2 signaling; and response to viral, bacterial, and parasitic infections, among others (Table 3, Supplemental File 3).

### 2.5. Dendritic Cells

#### 2.5.1. Top CSSG for Dendritic Cell

Top CSSG genes for dendritic cells include 510 genes (Supplemental File 1). Top enriched biological categories affected by chemical exposures are involved in the regulation of protein synthesis and processing (TRBP containing complex, protein folding, protein processing in endoplasmic reticulum, and others); immune response (leukocyte activation, cytokine signaling, VEGFA-VEGFR2 signaling, and response to viral infection); response to stress (response to toxic substance, response to oxidative stress, and fluid shear stress); mitochondria function (oxidation-reduction process, mitochondrion organization, aerobic respiration, and monocarboxylic acid metabolism); and SLIT and ROBO cascade, which may be involved in the regulation of cell migration and angiogenesis; and apoptosis (Table 1, Supplemental File 2).

#### 2.5.2. Genes Affected by Chemicals and COVID-19

A total of 623 genes were affected by COVID-19. Out of these 623 genes, 20 genes (3.2%) overlapped with the top dendritic cell-specific chemically sensitive genes (CSSG) (Figure 2). The top overlapping genes include, for example, genes encoding chaperones (HSPA5, FKBP5, HSP90AB1, and HSPA8); major histocompatibility complex protein (HLA-DQB1); TRIM22, which mediates interferon’s antiviral effects; EEF2; and PABPC1 genes involved in protein synthesis (Table 2). Metascape analysis showed that the top overlapping genes are involved in the response to viral infection (regulation of viral life cycle, viral entry into host cell, response to virus, response to interferon-gamma, and RNA degradation); antigen processing and presentation; P2X7 receptor signaling complex, which senses ATP released by dying cells and plays a role in inflammation; IL-18 signaling; and apoptotic signaling (Table 3, Supplemental File 3).

### 2.6. Top Sensitive Overlapping Genes in Multiple Cell Types

Eight genes were identified as the topmost genes sensitive to chemical exposures and COVID-19 in more than one cell type. They include genes involved in the regulation of protein synthesis (EEF2, EEF1A1, PABPC1, and TPT1), chaperones (HSPA5 and HSP90B1), and key players in interferon signaling cascade (ISG15 and STAT1).

Biological functions sensitive to chemical exposures across different cell types include cytokine signaling, VEGFA-VEGFR2 signaling, apoptotic signaling pathway, oxidation and reduction processes, protein synthesis, and folding. Biological functions most sensitive to chemical exposure and COVID-19 in more than one cell type include cytokine signaling as well as antigen processing and presentation.

## 3. Discussion

This study is the first to provide an overview of overlapping molecular mechanisms affected by COVID-19 and a broad range of chemical exposures. Previous research suggested that exposure to chemicals can increase COVID-19 disease severity [28,29,30]. The molecular mechanisms underlying the adverse effect of chemical exposures on COVID-19 progression remain mostly unknown. Our study allows the prediction of molecular mechanisms affected by chemical exposures, which may be responsible for severe progression of COVID-19.

Today, COVID-19 severity is linked to abnormal behavior of an innate immune system, characterized by a delayed IFN response and excessive inflammatory response [21,25].

IFN-mediated signaling plays a central role in anti-viral defense mechanisms [31]. Response to SARS-CoV-2 starts from the activation of pattern recognition receptors (PRRs) primarily in epithelial and endothelial cells, alveolar macrophages, NK cells, dendritic cells, and inflammatory monocyte-macrophages [21]. PRRs then activate the IFN production pathway via a cascade of adaptors and IRF proteins. IFNs bind to their specific receptors, leading to the phosphorylation of STAT1 and STAT2 by JAKs. This phosphorylation results in the formation of an IFN-stimulated gene factor 3 (ISGF3) complex containing STAT1, STAT2, and a transcription factor IRF9. Further translocation of ISGF3 to the nucleus results in its binding to IFN-stimulated response elements (ISREs) in promoters of IFN-stimulated genes and induction of their transcription.

IFN-stimulated genes are involved in many lines of antiviral defense, such as suppression of protein synthesis to prevent replication of pathogens, promotion of apoptosis of virus-infected cells via the p53 pathway, activation of major histocompatibility complexes I (MHC I, all IFNs) and II (MHC II, IFNγ) to increase antigen presentation, increase in immunoproteasome activity to boost the process of antigen loading to MHC I, suppression of angiogenesis, and activation of other immune cells. It is important to mention here that positive regulation of antigen presentation by IFNs results in activation of the adaptive branch of the immune system, as both T-cells and B-cells are activated by antigen presentation by dendritic cells and macrophages.

This brief overview of molecular mechanisms involved in the IFN I/II cascade and biological functions regulated downstream suggests that chemical exposures may increase the severity of COVID-19 owing to their interaction with IFN signaling cascade. Indeed, the majority of genes and enriched molecular pathways affected by both stressors, COVID-19 and xenobiotics, are relevant to IFN signaling. Key IFN signaling genes were identified as sensitive to COVID-19 and chemical exposures in all analyzed cells (not all of them are shown in tables in the text; see Supplemental File 1 for more detail). For example, IFN gamma receptor was among the top CSSG genes in monocytes and dendritic cells, while STAT1 was sensitive to COVID-19 and xenobiotics in monocytes, NK cells, and T-cells. Additionally, the IFN-signaling pathway was highly enriched in NK cells, and response to IFN-gamma was enriched in dendritic cells and T-cells. Similarly, biological categories regulated downstream of IFN were enriched in most cells, including protein synthesis, apoptosis, antigen processing and presentation, cell cycle, and angiogenesis pathway (VEGFA-VEGFR2).

It is interesting that, among genes sensitive to chemical exposures and COVID-19, we did not find genes involved in the regulation of IFN production in response to viral infection. In other words, our data suggest that IFN signaling is disrupted at some downstream steps, following IFN binding to their receptors. Emerging evidence demonstrates that SARS-CoV-2 has several strategies to suppress IFN signaling, including avoidance of detection by host PRRs and inhibition of the signal transduction mediated by PRRs and IFN receptors [23]. Our data suggest that SARS-CoV-2 and chemical exposures may synergistically impair the IFN signaling cascade, with the virus mostly suppressing the initial steps leading to IFN production, while xenobiotics affect the cascade starting from IFN binding to IFN receptors until transcription of IFN-stimulated genes.

Given that severe progression of COVID-19 is associated with a dysregulated inflammatory response, many studies suggested that SARS-CoV-2 may cause a “cytokine storm’’ responsible for increased morbidity and mortality [32,33,34,35]. Several studies attempted to identify inflammatory molecules associated with severe COVID-19 [25,36,37,38]. Although the profile of inflammatory markers is highly variable between patients, a general phenotype of severe COVID-19 is characterized by elevated IL-6, IL-8, IL-10, TNF-alpha, CCL2, CCL3, and CXCL8 [21]. All these genes were not members of the lists of the most chemically sensitive genes in all analyzed immune cells, except CCL2, which was among the top CSSG genes for monocytes. Thus, it is likely that chemical exposure does not play a significant role in promoting an excessive inflammatory response—a “cytokine storm”.

In our analysis, we observed the highest overlap (20%) between the top CSSG genes and genes affected by SARS-CoV-2 in monocytes. This observation suggests that the functions of monocytes may be compromised in COVID-19 patients exposed to high doses of various chemicals. Monocytes are professional antigen presenters providing the link between innate and adaptive branches of the immune system. A delayed response of T-cells and B-cells owing to disrupted antigen presentation by monocytes may constitute the molecular mechanism responsible for severe COVID-19 progression in patients exposed to high doses of chemicals. For example, antigen presentation is required for differentiation of effector T-cells: cytotoxic T-cells are critical for elimination of virally infected host cells; T helper cells are responsible for stimulation of antibody class switching in B-cells and activation/recruitment of macrophages, neutrophils, and other innate cells.

The approach used in our study is based on a high level of generalization, which masks the complexity and diversity of individual chemical–gene interactions. Additionally, the direction of change in gene expression was not considered when sensitivities of genes to chemical exposures were calculated. In fact, our previous study demonstrated that the absolute majority of highly chemically sensitive genes are induced and suppressed by an almost equal number of compounds [15]. Similarly, a high level of generalization does not allow accounting for individual differences in patients’ response to COVID-19, including differences determined by age, sex, race, comorbidities, and other factors. Future experimental studies are needed to validate our in silico predictions.

## 4. Materials and Methods

### 4.1. Sensitivity of Genes to Chemical Exposures

The unbiased approach for the identification of genes sensitive to chemical exposures was developed in our previous studies [15,27]. A database was created by extracting data on chemical–gene interactions from the Comparative Toxicogenomics Database (CTD) [39] (Figure 1A) using the following filtering criteria. First, data were extracted only from experiments that used high-throughput approaches for gene expression analysis. In addition, we selected data only from experiments that used human, rat, or mouse cells or tissue for gene expression analysis in in vitro and in vivo studies. Further, we removed from the database all genes that are not present in the genomes of all three species (human, rat, and mouse). The resulting database included 591,084 entries, each representing one CGI. At the next step, the number of CGIs was calculated for every gene to represent its sensitivity to chemical exposures (Figure 1B). Our previous study has shown that ranked sensitivities of genes to chemical exposures do not depend on the composition of chemicals used in original studies—sources of transcriptomic data [15], suggesting that our method provides unbiased results.

### 4.2. Sensitivities of Genes to Chemical Exposures Normalized for Expression in Different Cell Types

Normalized gene expression (NGE) values for human T-cells, B-cells, NK cells, dendritic, and monocyte cells were extracted from the Human Protein Atlas [40] (Figure 1C) and used to adjust the chemical sensitivity of genes for the level of expression in different immune cells. For this adjustment, CGI values were magnified by NGE values for corresponding cells (CSSGs = CGI × NGE) (Figure 1D) (Supplemental File 4). To select the top genes with the highest CSSG values for enrichment analysis, we used an approach of cutoff point identification in descriptive high-throughput -omics studies described in detail elsewhere [41]. In short, the cutoff point was identified as a point of transition from exponential to super-exponential phases of the curve of ranked CSSG values. The major assumption of that approach is that the small number of genes with high CSSG values dominate changes in biological processes and functions in response to chemical exposures.

### 4.3. Genes Affected by SARS-CoV-2 in Human Immune Cells

The Coronascape (coronascape.org) database was used as a source of information on gene expression changes in human tissues in response to COVID-19 exposures (Figure 1E). This database contains 390 datasets representing 29 tissues and cell lines collected from 22 individual studies. Only datasets where the effect of COVID-19 was analyzed in immune cells of human patients were used (Table 4). In vitro studies were excluded from the analysis.

### 4.4. Overlap of Top CSSG Genes with Genes Affected by COVID-19 

To identify genes that are sensitive to both chemicals and COVID-19, we overlapped the top CSSG genes for each cell type with genes affected in Coronascape datasets in the same cell types. Percent overlap was calculated as follows: % overlap = number of top CSSG genes/COVID-19-affected genes (Figure 2).

### 4.5. Enrichment Analysis

The top genes with high CSSG values for each cell type as well as genes affected by chemical exposures and COVID-19 in immune cells were analyzed using Metascape analysis (Figure 1F) with default settings [45]. For enrichment analysis, we selected only chemically sensitive genes affected in a specific cell type in more than one patient-specific COVID-19 dataset. Most of the discussion and conclusions of the manuscript are based on the analysis of biological categories enriched with a very high level of statistical significance: −log(*p*) > 16 for categories sensitive to chemical exposures, and −log(*p*) > 4 for categories sensitive to both xenobiotics and SARS-CoV-2. Significant categories (−log(*p*) > 2) with lower statistical significance are shown in Supplemental Files 2 and 3.

## 5. Conclusions

This study demonstrates that overlapping molecular mechanisms affected by a broad range of chemical exposures and COVID-19 are linked to IFN type I/II signaling pathways and to the process of antigen presentation. These pathways are essential components of the immediate response of innate immunity against viral infections. Synergistic impairment of these mechanisms by environmental factors and SARS-CoV-2 infection may affect the magnitude of the adaptive immune response and result in severe progression of COVID-19. Based on our data, we predict that exposures to various chemical compounds will predominantly impact the population of monocytes during the response against COVID-19. Our data do not provide any support of a hypothesis that chemical exposures may exacerbate a COVID-19-induced “cytokine storm”. The approach used in this study has many limitations. Future development of publicly accessible information-rich databases may help to improve the outcomes of in silico research. For example, information on sex, age, comorbidities, and others may add new layers of analysis of COVID-19-associated changes in molecular pathways. Similarly, information on doses and exposure protocols used in toxicological experiments will allow stratifying analysis in accordance with exposure scenarios.

## Figures and Tables

**Table 1:** Top ten biological functions most sensitive to chemical exposures in immune cells. Biological functions identified as most sensitive in more than one cell type are shown in bold.

	NK Cells (649 Top CSSG)	T Cells (558 Top CSSG)	Monocytes (409 Top CSSG)	B Cells (408 Top CSSG)	Dendritic Cells (510 Top CSSG)
1	mRNA catabolic process	**Ribosome, cytoplasmic**	Leukocyte degranulation	**Ribosome, cytoplasmic**	Regulation of expression of SLITs and ROBOs
2	**Cytokine signaling in immune system**	**Cytokine signaling in immune system**	Cellular responses to external stimuli	**VEGFA-VEGFR2 signaling pathway**	Leukocyte activation involved in immune response
3	**VEGFA-VEGFR2 signaling pathway**	**VEGFA-VEGFR2 signaling pathway**	**Cytokine signaling in immune system**	TRBP containing complex	**Oxidation-reduction process**
4	**Oxidation-reduction process**	**Oxidation-reduction process**	**VEGFA-VEGFR2 signaling pathway**	**Cytokine signaling in immune system**	**VEGFA-VEGFR2 signaling pathway**
5	Leukocyte activation involved in immune response	**Positive regulation of cell death**	**Positive regulation of cell death**	Nucleoside monophosphate metabolic process	Cytokine signaling in immune system
6	**Ribonucleoprotein complex biogenesis**	**Apoptotic signaling pathway**	**Apoptotic signaling pathway**	**Ribosome assembly**	TRBP containing complex
7	Protein processing in the endoplasmic reticulum	Leukocyte activation involved in immune response	Oxidation-reduction process	Regulation of translation	**Apoptotic signaling pathway**
8	**Regulation of apoptotic signaling pathway**	Ribonucleoprotein complex assembly	Nuclear receptors meta-pathway	Protein folding	Response to toxic substance
9	Protein folding	Regulation of cellular amide metabolic process	Cellular response to interleukin-12	**Apoptotic signaling pathway**	Mitochondrion organization
10	Regulation of cellular amide metabolic process	Epstein–Barr virus infection	Hemostasis	**Positive regulation of cell death**	**Positive regulation of cell death**

**Table 2:** Top ten genes sensitive to COVID-19 and chemical exposures in immune cells. Genes sensitive to both xenobiotics and SARS-CoV-2 in more than one cell type are shown in bold.

	NK Cells	T Cells	Monocytes	B Cells	Dendritic Cells
1	**STAT1**	**TPT1**	S100A8	MS4A1	HLA-DQB1
2	**ISG15**	**EEF1A1**	PLAC8	CD74	**HSPA5**
3	ACTB	**STAT1**	IFITM3	JCHAIN; IGJ	**ISG15**
4	GNLY	MX1	**PABPC1**	**HSPA5**	**PABPC1**
5	**EEF2**	**EEF2**	NAP1L1	CALR	**EEF2**
6	JAK1	**PABPC1**	S100A9	**HSP90B1**	TRIM22
7	SP100	EIF4B	**EEF1A1**	PDIA6	FKBP5
8	**PABPC1**	**ISG15**	**EEF2**	PDIA4	PLSCR1
9	**TPT1**	IL7R	CD14	CD79B	**HSP90AB1**
10	ISG20	PIM1	IFI6	CD79A	HSPA8

**Table 3:** Top ten biological functions most sensitive to chemical exposures and COVID-19 disease in immune cells. Biological functions sensitive to both xenobiotics and SARS-CoV-2 in more than one cell type are shown in bold.

	NK Cells (24 Gene Overlap)	T Cells (55 Gene Overlap)	Monocytes (124 Gene Overlap)	B Cells (65 Gene Overlap)	Dendritic Cells (20 Gene Overlap)
1	Regulation of multi-organism process	**Cytokine signaling in immune system**	Neutrophil degranulation	Regulation of multi-organism process	Regulation of viral life cycle
2	Interferon signaling	Chaperone-mediated autophagy	**Cytokine signaling in immune system**	Protein folding	**Antigen processing and presentation**
3	T cell mediated cytotoxicity	Translation factors	Regulation of multi-organism process	VEGFA-VEGFR2 signaling pathway	P2X7 receptor signaling complex
4	PID IL12 2PATHWAY	Regulation of multi-organism process	Defense response to other organisms	Translation factors	Viral entry into host cell
5	Protein methylation	**Antigen processing and presentation**	Activation of immune response	Regulation of myeloid cell differentiation	Response to virus
6	**Antigen processing and presentation**	Regulation of hemopoiesis	Apoptotic signaling pathway	B cell activation	Translation factors
7	Translation factors	I-kappaB kinase/NF-kappaB signaling	Response to inorganic substance	**Cytokine signaling in immune system**	Response to interferon-gamma
8	Negative regulation of binding	Epstein–Barr virus infection	Regulation of cytokine production	Chaperone-mediated protein folding	Negative regulation of the cellular component organization
9	Homotypic cell–cell adhesion	Response to interferon-gamma	Pertussis	Translation	RNA degradation
10	Allograft rejection	H2AX complex, isolated from cells without IR exposure	**Antigen processing and presentation**	Interaction with symbiont	Negative regulation of intrinsic apoptotic signaling pathway

**Table 4:** Total number of COVID-19 data sets for each cell type extracted from Coronascape and used in this study. Each dataset corresponds to one patient.

Cell Type	Number of Datasets	Genes Affected by COVID-19 in More than One Dataset	Sources
Dendritic cells	14	53	[42]
NK cells	19	81	[42,43]
B-cells	23	248	[42,43,44]
Monocytes	32	278	[42,43]
T-cells	35	116	[42,43]

## Data Availability

All data used in this study are available from published sources and public databases. Information on genes’ sensitivity to chemical exposures was published previously [15,27]. Normalized gene expression (NGE) values for human immune cells are available from the Human Protein Atlas [40]. Information on gene expression changes in human tissues in response to COVID-19 exposures is available from the Coronascape database.

## Supplementary Materials

The following are available online at https://www.mdpi.com/article/10.3390/ijms222212474/s1.
